# Abyssinone V-4' Methyl Ether Isolated from* Erythrina droogmansiana* (Leguminosae) Inhibits Cell Growth and Mammary Glands Hyperplasia Induced in Swiss Mice by the 7,12-Dimethylbenz(a)anthracene

**DOI:** 10.1155/2018/7959068

**Published:** 2018-07-11

**Authors:** Alain Brice Tueche, Stephane Zingue, Edwige Nana Tchoupang, Telesphore Nanbo Gueyo, Abel Joël Gbaweng Yaya, Amstrong Nang Njuh, Dieudonnée Mireille Ntsa, Dieudonné Njamen

**Affiliations:** ^1^Department of Biological Sciences, Faculty of Science, University of Maroua, P.O. Box 814, Maroua, Cameroon; ^2^Department of Life and Earth Sciences, Higher Teachers' Training College, University of Maroua, P.O. Box 55, Maroua, Cameroon; ^3^Department of Animal Biology and Physiology, Faculty of Science, University of Yaoundé 1, P.O. Box 812, Yaounde, Cameroon; ^4^Centre for Research on Medicinal Plants and Traditional Medicine, Institute of Medical Research and Medicinal Plants Studies, Yaounde, Cameroon

## Abstract

There is a long standing interest in the identification of medicinal plants and derived natural products for developing cancer therapeutics. The present study was designed to evaluate the* in vitro *and* in vivo* antiproliferative effects of Abyssinone V-4' methyl ether (AVME) on breast tissue of mice. The cytotoxicity of AVME was evaluated using MTT assay in four cancer cell lines (DU145, PC3, HepG2, and MCF-7). Further, a protective effect of AVME was evaluated on 7,12-dimethylbenz(a)anthracene- (DMBA-) induced breast tumor in Swiss mice. Incidence, burden, volume, and histological analysis of mammary tumors were measured. As a result, AVME inhibits DU145, PC3, HepG2, and MCF-7 cells growth.* In vivo*, no tumor was detected in mice from the normal group as compared to those of DMBA group. Moreover, AVME inhibits the DMBA-induced mammary glands hyperplasia in mice at the dose of 10 mg/kg, evidenced by a decrease of tumor incidence, tumor weight, and volume as well as a protective effect against the lobular alveolar hyperplasia. Taken all together, these results suggest that Abyssinone V-4' methyl ether is endowed with antitumor properties and could be a source of traditional medicine which deserves to be more elucidated and explored in the foreseeable future.

## 1. Introduction

Cancer is a heterogenous condition where normal cells take up a malignant phenotype, proliferating indefinitely and uncontrollably, thereby leading to a destruction of the original tissue [1] and later advances in a multistep process with various hallmarks including rapid proliferation, resisting cell death, neoangiogenesis, local invasion, and remote metastasis [2]. It is an important cause of death worldwide, posing a major threat to public health. More than 1.1 million people are diagnosed of cancer each year; among them almost 410,000 deaths are registered. According to Plummer* et al. *[3], over 8 million deaths can be linked with cancer. The World Health Organization (WHO) fears that, by 2030, cancer deaths may increase by up to 50%, thereby reaching about 15.000.000. There are many types of cancers, among which breast cancer is the leading cause of death in women with 50,000 new cases per year [4]. In Cameroon, according to the Globocan estimation, among women, breast cancer is the most common before the cervical cancer with 27.9 per 100,000 cases [1]. Globally, about one million women are detected with this condition annually, resulting in over 400.000 deaths from the pathology [5]. Several factors have been correlated with breast cancer; however, estrogen-dependent cancers are the most prevalent hormone-related breast cancers with 50-80% of all breast tumors and their proportion increases with age [6].

In recent years, four main medical interventions, radiotherapy, chemotherapy, immunotherapy, and hormone therapy, constitute the frontline therapies for breast cancer, surgery for advanced stages of the disease [7, 8]. However, cancer management is plagued with many limitations. Particularly for Africa, treatment cost is high, and centers for diagnosis and management are limited. These, coupled with drug resistance and systemic toxicity of current chemotherapeutic molecules, pile up the difficulties faced using the different anticancer therapies [9, 10]. Faced with these difficulties, the quest for new anticancer molecules which are readily available, effective, and less expensive becomes a serious issue if these problems have to be overcome. Plants which are easily exploited give hope and make up a huge variety of anticancer substances with a wide variety of bioactive compounds [11]. Among these metabolites, there are abyssinones which are prenylated flavonoids. These molecules have gained attention since they are endowed with a large range of activities such as aromatase inhibitory, antioxidant, and cytotoxic activities [12, 13]. The aim of the present study is to bring a significant contribution to the fight against cancer, by evaluating the* in vitro* and* in vivo* antiproliferative effects of Abyssinone V-4' methyl ether (AVME) isolated from* Erythrina droogmansiana* (Leguminosae). For this to be done, the ability of AVME to inhibit cancer cells growth* in vitro* and to prevent a 7,12-dimethylbenz(a)anthracene-induced breast tumor in female mice was evaluated.

## 2. Materials and Methods

### 2.1. Chemicals and Reagents

The following products were acquired from Gibco/Invitrogen (Karlsruhe, Germany): penicillin, streptomycin, fetal bovine serum, glutamax, and the 2-[4-(2-hydroxyethyl)piperazin-1-yl]ethane sulfonic acid (HEPES). The 3-(4,5-dimethylthiazol-2-yl)-2,5-diphenyltetrazolium bromide (MTT) and 7,12-dimethylbenz(a)anthracene (DMBA) (purity ≥ 95%) were acquired from Roche Diagnostics (Penzberg, Germany) and Sigma-Aldrich (Stanford, Germany), respectively.

### 2.2. Plant Material

#### 2.2.1. Collection and Authentication

Root barks of* Erythrina droogmansiana *T. Durand (Fabaceae) were collected in Nkomekoui-Yaounde (Centre Region of Cameroon) on 21 August 2010 at about 8:00 A.M. and identified at the Cameroon National Herbarium (CNH) (Voucher specimen N°4261/SRFK) by the botanist M. Victor Nana.

#### 2.2.2. Preparation of the Extract

A total of 1,200 g of air-dried and pulverized root barks of* E. droogmansiana* was macerated in 5 liters of ethyl acetate 99.4% for 2 days at room temperature. Then, 150 g (12.5 %) of crude ethyl acetate extract was obtained after filtration through a Whatman n° 4 filter paper and evaporation of the ethyl acetate using a rotary evaporator.

#### 2.2.3. Isolation of Abyssinone V-4' Methyl Ether

One hundred grams of the obtained extract was subjected to chromatography over silica gel (200-425 mesh particle size) packed in n-hexane. Gradient elution was done using n-hexane, hexane-ethyl acetate, and ethyl acetate-methanol in increasing polarity (hex 100%, hex-ethyl acetate: 90-10, 80-20, 70-30, 50-50, 30-70, 20-80, ethyl acetate 100%; ethyl acetate-MeOH: 90-10, 80-20, 70-30, 50-50, 30-70, 20-80, and MeOH 100%) to give 7 series of fractions mixed base on their TLC profile. Repeated column chromatography with hexane-ethyl acetate (90:10) yielded to YG_4_ and other compounds. The structures have been elucidated using spectral methods (MS, NMR, and element analysis). The compound YG_4_ was obtained as a white powder (500 mg) and showed a [M]^+^ at m/z 422.2094 corresponding to molecular formula (C_26_H_30_O_5_). This compound was identified as Abyssinone V-4' methyl ether (Table 1). The presence of a flavanone skeleton was evident from the ^1^HNMR spectra at *δ* 5.27 (1H. br), 2.68 (1H, dd,* J* = 2.8 and 17.2 Hz) and *δ* 3.06 (1H. dd.* J* = 13.2 and 17.2) corresponding to the H-2 and H-3 protons of the C-ring of flavanones, respectively. From the ^13^CNMR spectra, the presence of signal at 79.3 and 42.5, respectively, indicated the C-2 and C-3 of the C- ring of flavanones. The ^1^H and ^13^CNMR spectra data of this compound were compared to those of the same compound previously isolated [14].

### 2.3. In Vitro Experiment

#### 2.3.1. Cell and Cell Culture

The following cancer cell lines, MCF-7 (human ER-positive breast adenocarcinoma cells), HepG2 (human liver hepatocellular carcinoma), and DU145 and PC3 (human androgens independent prostate carcinoma) cells, were acquired from LGC Promochem (Wesel, Germany).

Culture of HepG2 cells was done using Dulbecco's Modified Eagle Medium (DMEM) supplemented with 10% of fetal bovine serum (FBS). RPMI 1640 medium supplemented with 10% fetal bovine serum (FBS) was used for the growth and subculture of MCF-7, DU145, and PC3 cells. All cell cultures were also supplemented with penicillin 100 U/mL, streptomycin 100 *μ*g/mL, and HEPES 10 mM. The cell cultures were maintained at 37°C in a CO_2_ 5% humidified atmosphere and pH 7.4. After every other day, 90% of the supernatant was replaced with fresh medium during the cells passage. The number of viable cells was assessed using the trypan blue method and Neubauer chamber was used to assess the number of viable cells and cell count, respectively, prior to performing all experiments.

#### 2.3.2. Treatment of Cells in Culture with Abyssinone V-4' Methyl Ether

Abyssinone V-4' methyl ether (AVME) was freshly dissolved in DMSO and tested in final concentrations ranging from 0.5 to 20 *μ*M. Controls remained untreated. In all* in vitro* experiments, treated tumor cell cultures were compared to nontreated control cultures of the same passage and cell numbers per well.

#### 2.3.3. Tumor Cell Growth

The 3-(4,5-dimethylthiazol-2-yl)-2,5-diphenyltetrazolium bromide (MTT) dye reduction assay was used to assess cell growth. Treated versus nontreated tumor cells (100 *μ*l, 1 × 10^4^ cells/ml) were seeded onto 96-well tissue culture plates. After 24, 48, and 72h, MTT (0.5 mg/ml) was added for an additional 4 h. Thereafter, cells were lysed in a buffer containing 10% SDS in 0.01 M HCl. The plates were incubated overnight at 37°C, 5% CO_2_. Absorbance at 570 nm was determined for each well using a microplate enzyme-linked immunosorbent assay (ELISA) reader. Each experiment was done in triplicate and repeated 3 times. Results are expressed as mean cell number after subtracting background absorbance, and results are expressed as mean cell number.

### 2.4. In Vivo Experiment

#### 2.4.1. Animals

Experimental female Swiss mice, aged 25-31 days (7-10 g) used in this study, were obtained from the breeding facility of the Laboratory of Biochemistry, University of Yaounde I (Cameroon). Animals were housed in plastic cages at room temperature. They had free access to water and a standard soy-free mouse diet with the following composition: corn (36.7%), bone flour (14.5%), wheat (36.6%), fish flour (4.8%), crushed palm kernel (7.3%), sodium chloride (0.3%), and vitamin complex (Olivitazol®- 0.01%).

#### 2.4.2. Ethical Consideration

The Cameroon Institutional National Ethic Committee approved the housing of animals and all experiments. This committee adopted all procedures recommended by the European Union on the protection of animals used for scientific purposes (CEE Council 86/ 609; Reg. no. FWA-IRD 0001954).

#### 2.4.3. DMBA-Induced Breast Tumor in Mice

Eighteen (18) mice were acclimatized for 20 days and randomly assigned at age of 45-51 days into 3 groups of 6 animals in each. The breast tumor was induced on the 1^st^ day of experiment by a single dose of DMBA (100 mg/kg,* per os*) dissolved in 1 ml of olive oil to all the experimental groups (12 mice) except control group that received olive oil only. They received the following treatment schedule: the groups control (normal mice) and DMBA (negative control) received distilled water as vehicle. The last group was treated with AVME at the dose of 10 mg/kg. Fifty-six days after the administration of DMBA, all treatments were administered* i.p.* once daily at about 11:00 A.M. and continued in the same way until 28 days. Every week, experimental mice were weighed and palpated twice a week to check the development of mammary tumors from the first day of acclimatization until the end of the experiment. Tumorous latency (time of tumor appearance) was recorded. All animals that died during the experiment or those that became moribund were sacrificed and autopsy was carried out. At the end of the first month of treatment, all survivors were sacrificed after a 12 h overnight nonhydric fasting. Two blood samples were collected for each animal: one in an anticoagulant (EDTA) tube for hematological analysis and the other in a dry tube and centrifuged at 600 × g for 15 min at 4°C for biochemical analysis. The skin was also dissected out to expose mammary tumors and all tumors were removed, counted, and weighed. The size of tumors was measured using a 1 mm precisions sliding caliper (IGAGING®). Afterward, the tumorous incidence of different groups was recorded and the formula from Faustino-Rocha et al. [15] (length × weight × height ×  *π*/6) was used to calculate the tumor volume. Estrogen target organs were also removed and weighed. All these organs were fixed in 10% neutral formalin solution for histomorphology.

### 2.5. Histological Analysis

Mammary glands, uterus, and vagina were dehydrated by a series of ethanol solution and embedded in paraffin blocks. Microtomy resulted in 5 *μ*m sections which were stained with hematoxylin and eosin. An Axioskop 40 microscope connected to a computer was used to determine histomorphological changes. The image was transferred using MRGrab1.0 and Axio Vision 3.1 software (Zeiss Hallbermoos, Germany). Atlas and histologic classification of tumors of rat mammary gland from Russo and Russo [16] aided in this study.

### 2.6. Biochemical and Hematological Analysis

Regarding biochemical analysis, the liver enzyme aspartate transaminase (AST) activity and creatinine level were measured using reagent kits from Fortress Diagnostics Limited (Muckamore, United Kingdom).

Different hematological parameters were also evaluated using a MINDRAY BC-2800 Auto Hematology Analyzer from Shenzhen Mindray Bop-Medical Electronics Co., Ltd. These are white blood cell (WBC) count, lymphocytes, monocytes, granulocytes, red blood cell (RBC) count, and platelets.

### 2.7. Statistical Analysis

Results are presented as means ± standard deviation (SD) for each experimental group. Statistical analysis of data with Sigma plot software version 11.00 was realised using the one-way analysis of variance (ANOVA) followed by Dunnett's post hoc test for multiple comparisons. Statistical significance of differences was considered at a p value < 0.05.

## 3. Results

### 3.1. Tumor Cell Growth

**Figure 1:**
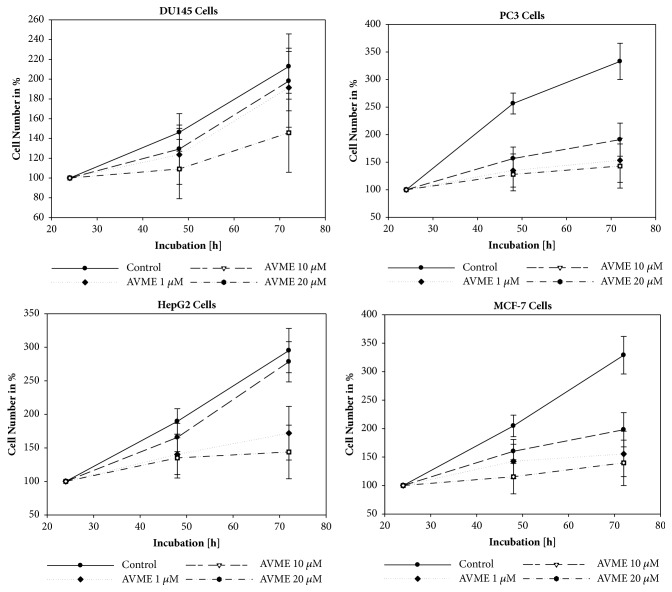
Growth of DU145, PC3, HepG2, and MCF-7 carcinoma cells treated with different concentrations of Abyssinone V-4' methyl ether (AVME) after 24, 48, and 72 hours. Controls remained untreated (n = 3).

Abyssinone V-4' methyl ether (AVME) showed a concentration-dependent inhibition of DU145, PC3, HepG2, and MCF-7 cells growth. It was also observed that the degree of cytotoxic activity increased time dependently. However, Figure 1 shows only the results for AVME at optimum concentrations. The most significant activity was observed at the final concentrations of 10 and 20 *μ*M in all tested cells.

### 3.2. Effects on Body Weight

**Figure 2:**
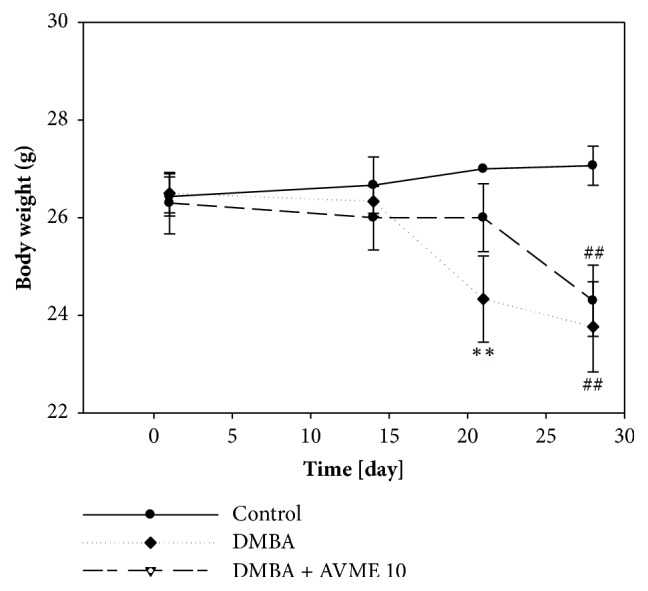
Body weight evolution after 4 weeks of treatment. Control = normal mice treated with distilled water; DMBA = negative control treated with distilled water; DMBA + AVME 10 = animals treated with the Abyssinone V-4' methyl ether at the dose of 10 mg/kg. All groups excepting the normal group (control) received an intragastric dose of DMBA at the dose of 100 mg/kg. Data are represented as mean ± SD (n = 5). *∗∗*p < 0.01 as compared to negative control (DMBA); ^##^p < 0.01 as compared to normal group.

The effects of Abyssinone V-4' methyl ether (AVME) were evaluated after 4 weeks of treatment on body weight (Figure 2). It can be observed that the growth of all normal mice was continuous throughout the treatment. However, from the 20^th^ day until the end of the experiment, a significant (p < 0.01) decrease of the body weight in DMBA mice and those treated with AVME (10) was observed. Animals treated with AVME presented a significant (p < 0.01) lower body weight as compared to the DMBA control group at the 21^th^ day.

### 3.3. Effects on Mammary Tumors

Table 2 presents data related to chemopreventive activity of Abyssinone V-4' methyl ether (AVME) on mammary glands alteration after 28 days of treatment. No palpable tumor was observed among all the groups 3 months after DMBA (100 mg/kg) exposition. However, the animals from the normal group presented no localized tumor, while the animals of the DMBA group presented 60% of localized mammary tumors (noodles). Animals treated with AVME at the dose of 10 mg/kg showed a significant (p < 0.001) reduction in tumor incidence (20%), tumor volume (6.8 ± 0.5 cm^3^ versus 15.32 ± 3.01 cm^3^ in DMBA group), and an inhibition related to the average tumor weight of 53.84% as compared to DMBA animals.

### 3.4. Histomorphological Analysis of Estrogen Target Organs

**Figure 3:**
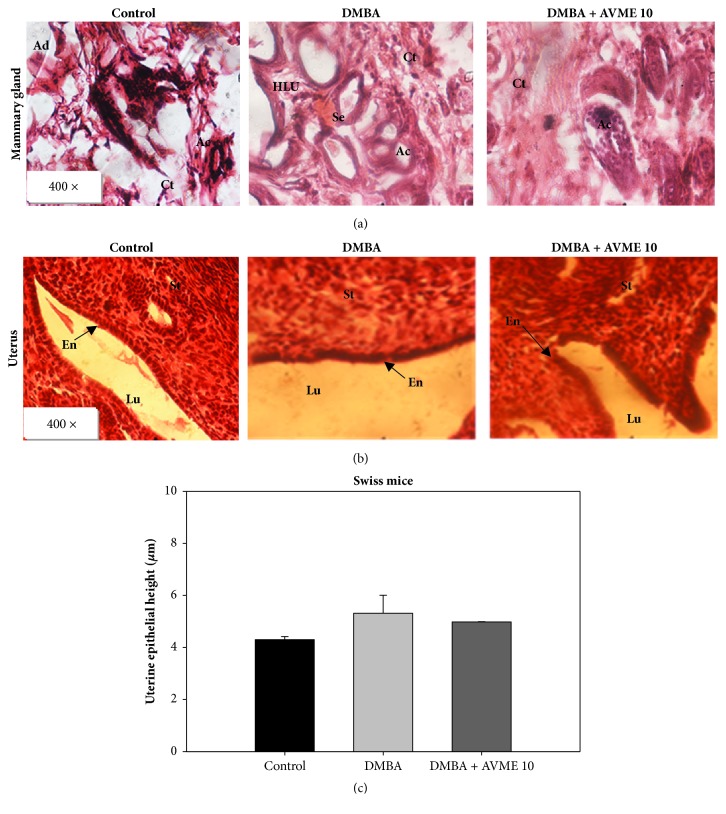
Effects of Abyssinone V-4' methyl ether on microphotographs H&E 400× of mammary glands (**a**), uterine (**b**), and graphic representation of uterine epithelial height (**c**) after 28 days of treatment. NOR = normal mice treated with distilled water; DMBA = negative control treated with water; DMBA + AVME 10 = animals treated with the Abyssinone V-4' methyl ether at the dose of 10 mg/kg. All groups except the normal group (control) received an intragastric dose of DMBA at the dose of 100 mg/kg. Data are represented as mean ± SD (n = 5). At = adipose tissue; Se = eosinophil secretion, L = lobular; HLU = hyperplastic lobular unit; St = stroma, Lu = lumen of uterus, Ge = germinal epithelium, En = endometrium, and Ct = conjunctive tissue.

The mammary gland microarchitectures of mice belonging to the normal group showed normal acini surrounded by a small amount of fibrous conjunctive tissue (Figure 3(a)). Mice that received only DMBA presented mammary gland carcinoma, evidenced by a diminution of the conjunctive tissue as well as a severe hyperplasia of mammary lobules and dilated ducts filled with tumoral cells. Further, in animals treated with AVME (10 mg/kg), a quasi-normal histoarchitecture of mammary glands with low cellular proliferation and low ductal dilation was observed. A nonsignificant increase of uterine epithelial height was observed in animal that received DMBA (Figures 3(b) and 3(c)).

**Figure 4:**
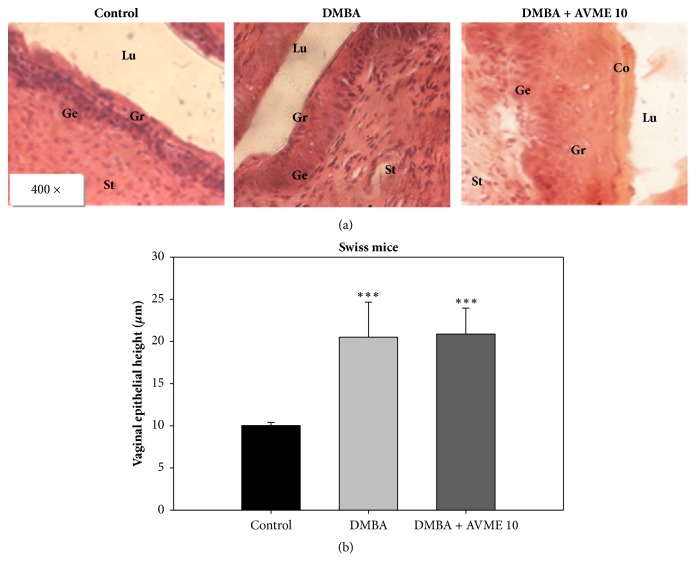
Effects of Abyssinone V-4' methyl ether on microphotographs H&E 400× of vagina (**a**) and graphic representation of vagina epithelial height (**b**) after 28 days of treatment. Control = normal mice treated with distilled water; DMBA = negative control treated with water; DMBA + AVME 10 = animals treated with the Abyssinone V-4' methyl ether at the dose of 10 mg/kg. All groups except the normal group (control) received an intragastric dose of DMBA at the dose of 100 mg/kg. Data are represented as mean ± SD (n = 5). *∗∗∗*p < 0.001 as compared to negative control. St = stroma, Co = stratum corneum, Gr = stratum granulosum, Lu = lumen of vagina, and Ge = stratum germinativum.

Moreover, a significant (p < 0.001) increase in the vaginal epithelial height was observed with the mice that received DMBA (Figure 4).

### 3.5. Effects of Abyssinone V-4' Methyl Ether on Relative Wet Weight of Organs

The relative weights of various organs following 28 days of treatment with Abyssinone V-4' methyl ether are depicted on Table 3. Between the normal and DMBA treated groups, no significant difference in the weight of various organs was observed, except for uterine wet weight that increases (p < 0.01) and spleen wet weight that decreases (p < 0.05), respectively. No significant change was found between DMBA control mice and AVME 10 treated mice in all assessed organ wet weights.

### 3.6. Effects on Some Biochemical and Hematological Parameters

Table 4 depicts the effects of Abyssinone V-4' methyl ether on some biochemical and hematological parameters. Mice that received only DMBA presented a significant increase in the AST activity (p < 0.05), creatinine (p < 0.05), granulocyte (p < 0.05), and platelets (p < 0.01) levels, while a significant (p < 0.01) decrease of hemoglobin level was found as compared to normal mice (Control). AVME at the dose of 10 mg/kg showed a significant decrease in the AST activity (p < 0.05), creatinine (p < 0.01), and granulocyte (p < 0.05) levels as compared to DMBA mice.

## 4. Discussion

Breast cancer is the second most common malignancy worldwide, and the first among women. Accounting for 23% of new cancer cases worldwide, breast cancer is considered to have a relatively good prognosis if early diagnosed and treated. In Cameroon appropriately, the median survival rate after five years is still weak (around 40%) as compared with developed countries. In addition, 57.9% of breast cancer patients are under 50 years, and 28.5% are less than 40 years [17]. Due to the high frequency with which breast cancer reaches the human population, the quest for effective and less expensive medical treatment becomes a serious need. Hence, novel molecules are continuously being identified and developed from medicinal plants, which in general are less toxic and have presented effective results [11]. To contribute to the quest for safer anticancer molecules the* in vitro* and* in vivo* antiproliferative properties of Abyssinone V-4' methyl ether isolated from* Erythrina droogmansiana* (Leguminosae) were explored. Regarding our* in vitro* antitumoral assays, one of our most important results shows that AVME has antiproliferative effects in all tested cell lines at the optimal concentration of 20 *μ*M. Of note, the MTT assay measures the ability of a test substance to cause cell death by alteration of one or more cellular functions through the evaluation of cell proteins production [18]. These effects suggest that AVME is endowed with anticancer properties and corroborate many studies which described flavonols as cytotoxic* in vitro* [19, 20].

For* in vivo* experiment, the protective effects of AVME on 7,12-dimethylbenz(a)anthracene- (DMBA-) induced breast tumors in mice were assessed. DMBA is a polycyclic aromatic hydrocarbon well-known potent environmental carcinogen which has been used to induce carcinogenesis in the mammary gland or skin of experimental rodents such as rat and mouse [16, 21]. In this study, DMBA was administered at a single dose of 100 mg/kg BW by intragastric gavage to Swiss mice aged between 45 and 50 days to induce mammary tumors. Due to the active proliferation of the terminal ducts in breast tissue for rodents in this range of age, they become very susceptible to carcinogens and tumor development [22]. Experimental animals that received only DMBA developed large tumors after 12 weeks of study, whereas normal animals did not exhibit tumors. This result is in agreement with studies of Minari and Okeke [23] that found similar results with DMBA in female Swiss mice after 6 weeks. The significant reduction in tumor volume and average tumor weight observed with AVME at the dose of 10 mg/kg suggest a protective effect of this compound on the mammary tumorigenesis. These effects could be explained by the ability of AVME to inhibit breast cancer cell growth, as observed* in vitro *in this study. This is in agreement with the previous studies showing the antiproliferative effects of herbal polyphenols, in various human cancer cell lines [24, 25]. Of note, breast tissue is a well-known target of flavonols, which could explain the observed cancer protective potential of AVME, since they are proapoptotic and are endowed with cell cycle arrest property [26]. Results obtained on histopathological examination of the mammary gland sections are in line with those observed in tumor volume and average tumor weight. In fact, the evidence of DMBA-induced lobular alveolar hyperplasia in the mammary gland histological sections of untreated mice suggests a neoplastic transformation, which is a hallmark of DMBA-induced tumor in rodents [22, 27]. This result is in accordance with several observations, which showed that DMBA alters the normal process of mammary gland differentiation of terminal ducts to alveoli and lobules [24, 25, 28]. The mice treated with AVME exhibited a quite-normal histological sections as compared to the* in situ* carcinoma observed in DMBA control animals. This strongly suggests a protective effect of AVME in DMBA-induced cell proliferation in the breast tissue of mice.

Abyssinones are prenylated flavanones existing in plant of the genus Erythrina well known to exhibit diverse biological activities including* in vitro* anticancer activities [12, 13]. In fact, Samaga et al. [29] showed that Abyssinones I and II triggered apoptosis by upregulation of p53 and Bax, while they downregulated Bcl-2. According to the same authors, Abyssinones I and II induced apoptosis through mitochondrial pathway releasing cytochrome c and Apaf-1 into cytosol, associated with activation of caspase-3. In addition, Kuete et al. [30] demonstrated the antiproliferative potential of Abyssinone IV. This later also induced apoptosis in morphologic variations in human leukemic lymphoblasts (CCRF-CEM cells) mediated by the loss of mitochondrial membrane potential (MMP) and increase in ROS production. These observations support the ability of abyssinones to trigger apoptosis via mitochondrial pathway by activation of caspase-3. Obviously, AVME could use similar mechanisms to exhibit its antitumor activities observed in this study.

As far as toxicological status of animals is concerned, relative weight of organs is a good indicator of toxic effects of tested substances [31]. In this study, a significant increase in the uterine wet weight was observed; however endometrium height was unchanged, suggesting that AVME is not a threat for endometrial cancer. In fact, this observation is in accordance with the previous ones, which reported that AVME is endowed with estrogenic/antiestrogenic properties in ovariectomized rats [32, 33]. Furthermore, an increase of the aspartate transaminase activity and creatinine level in DMBA control group was observed. This could be explained by toxic effects of DMBA, which is able to induce hemorrhagic injuries in different organs such as liver and kidney [34]. Nevertheless, AVME prevented the DMBA-induced liver and kidney injuries in mice.

## 5. Conclusion

In summary, Abyssinone V-4' methyl ether inhibits DU145, PC3, HepG2, and MCF-7 cells growth. Furthermore, AVME inhibits the DMBA-induced mammary glands hyperplasia in mice at the dose of 10 mg/kg, evidenced by the decrease of tumor incidence, tumor weight, and volume as well as a protective effect against the lobular alveolar hyperplasia. Taken all together, these results suggest that Abyssinone V-4' methyl ether is endowed with antitumor properties and could be a source of traditional medicine which deserves to be more elucidated and explored in the foreseeable future.

## Figures and Tables

**Table 1:** General information on Abyssinone V-4'-methyl ether isolated from *Erythrina droogmansiana*.

**Chemical names**	**Crystal color**	**Structure, molecular weight, and formula**
Abyssinone V-4' methyl ether 4H-1-benzopyran-4-one, 2,3-dihydro-5,7-dihydroxy-2-[4-methoxy-3,5-bis(3-methyl-2-buten-1-yl)phenyl]	White	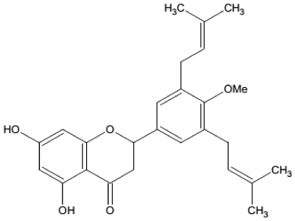 Molecular weight = 422.53 M Molecular formula = C_26_H_30_O_5_

**Table 2:** Breast cancer chemopreventive activity of Abyssinone V-4' methyl ether after 28 days of treatment.

**Items**	**Control**	**DMBA**	**DMBA + AVME 10**
Number of mice with tumours/total rats	0/6	3/5	1/5
Localized tumor incidence (%)	0	60 ^###^	20*∗∗∗*
Average tumor weight (g/kg)	-	1.04 ± 03.4	0.48 ± 0.92*∗∗∗*
% Inhibition related to tumor weight	-	-	53.84
Total tumor burden (g)	0	3.92	1.67
% Inhibition related to tumor burden	-	-	57.39
Volume (cm^3^)	-	15.32 ± 3.01	6.8 ± 0.5*∗∗*

NOR = normal mice treated with distilled water; DMBA = negative control treated with water; DMBA + AVME 10 = animals treated with the Abyssinone V-4' methyl etherat the dose of 10 mg/kg. All groups except the normal group (control) received an intragastric dose of DMBA at the dose of 100 mg/kg. Data are represented as mean ± SD (n = 5). *∗∗*p < 0.01 and *∗∗∗*p < 0.001 as compared to negative control; ^###^p < 0.001 as compared to normal group.

**Table 3:** Effects of Abyssinone V-4' methyl etheron relative wet weights of various organs following 28 days of treatments.

**Organs**	**Control**	**DMBA**	**DMBA + AVME 10**
Uterus	1539.63 ± 372.67	2048.19 ± 184.88##	2165.28 ± 180.21##
Liver	40621.32 ± 951.37	36907.63 ± 373.71	44855.91 ± 490.77
Lungs	8426.95 ± 422.95	10602.41 ± 888.25	12583.0 ± 812.51
Spleen	4322.87 ± 201.90	2851.40 ± 380.63#	3420.04 ± 609.28##
Adrenals	675.79 ± 76.03	441.76 ± 69.19	571.00 ± 90.5
Kidneys	10004.61 ± 801.91	11405.62 ± 714.75	11153.19 ± 643.13
Femur	2572.63 ± 553.63	2428.19 ± 81.83	2669.94 ± 124.11
Brain	2580.84 ± 111.1	3052.21 ± 171.22	2535.22 ± 127.81
Ovaries	408.40 ± 70.44	321.28 ± 71.22	546.60 ± 67.50

Control = normal mice treated with distilled water; DMBA = negative control treated with distilled water; DMBA + AVME 10 = animals treated with the Abyssinone V-4' methyl etherat the dose of 10 mg/kg. All groups except the normal group (control) received an intragastric dose of DMBA at the dose of 100 mg/kg. Data are represented as mean ± SD (n = 5). ^#^p < 0.05 and ^##^p < 0.01 as compared to normal group.

**Table 4:** Effects of Abyssinone V-4' methyl etheron some biochemical and hematological parameters following 28 days of treatment.

**Item**	**Control**	**DMBA**	**DMBA + AVME 10**
**Biochemical parameters**			
AST	37.2 ± 1.26	42.6 ± 2.13^#^	28.6 ± 1.78*∗*
Creatinine (g/L)	0.8 ± 0.06	1.3 ± 0.10^#^	0.36 ± 0.08*∗∗*

**Hematological parameters**			
WBC (×10^3^*µ*L^−1^)	7.42 ± 2.67	7.4 ± 2.48	8.7 ± 0.72
Lymphocytes (%)	61.1 ± 4.32	57.11 ± 4.91	64.5 ± 5.69
Monocytes (%)	11.4 ± 3.3	11.2 ± 0.04	12.67 ± 2.08
Granulocytes (%)	25.1 ± 5.5	32.1 ± 4.59^#^	23.23 ± 4.28*∗*
Hemoglobin	171.1 ± 6.99	138.2 ± 9.35^##^	151.66 ± 16.17
RBC (×10^3^*µ*L^−1^)	9.2 ± 0.051	8.48 ± 0.35	9.13 ± 1.08
Platelets (×10^3^*µ*L^−1^)	453 ± 10.42	959.2 ± 11.07^##^	833.33 ± 294.65^##^

Control = normal mice treated with distilled water; DMBA = negative control treated with distilled water; DMBA + AVME 10 = animals treated with the Abyssinone V-4' methyl etherat the dose of 10 mg/kg. All groups except the normal group (control) received an intragastric dose of DMBA at the dose of 100 mg/kg. Data are represented as mean ± SD (n = 5). *∗*p < 0.05. *∗∗*p < 0.01 as compared to negative control; ^#^p < 0.05 and ^##^p < 0.01 as compared to normal group.

## Data Availability

The data used to support the findings of this study are included within the article.

## Abbreviations

AVME: Abyssinone V-4' methyl ether
AST: Aspartate transaminase
BW: Body weight
CC_50_: Cytotoxic concentration for 50% of the cells
CCRF-CEM: Morphologic variations in human leukemic lymphoblasts
DMEM: Dulbecco's Modified Eagle Medium
DMBA: 7,12 dimethylbenz(a)anthracene
DMSO: Dimethylsulfoxide
ER: Estrogen receptor
FBS: Fetal bovine serum
HEPES: 4-(2-hydroxyethyl)-1-piperazineethanesulfonic acid
Hb: Hemoglobin
Ht: Hematocrit
NHC: National Herbarium of Cameroon
RBC: Red blood cell
RPMI: Roswell Park Memorial Institute medium
SD: Standard deviation.
